# Continuous Exposure of Breast Cancer Cells to Tamoxifen Upregulates GPER-1 and Increases Cell Proliferation

**DOI:** 10.3389/fendo.2020.563165

**Published:** 2020-09-30

**Authors:** Luis Molina, Felipe Bustamante, Alexander Ortloff, Iraidi Ramos, Pamela Ehrenfeld, Carlos D. Figueroa

**Affiliations:** ^1^ Facultad de Medicina y Ciencia, Universidad San Sebastián, Puerto Montt, Chile; ^2^ Laboratory of Cellular Pathology, Institute of Anatomy, Histology and Pathology, Universidad Austral de Chile, Valdivia, Chile; ^3^ Centro Interdisciplinario de Estudios del Sistema Nervioso (CISNe), Universidad Austral de Chile, Valdivia, Chile; ^4^ Departamento de Ciencias Veterinarias y Salud Pública, Facultad de Recursos Naturales, Universidad Católica de Temuco, Temuco, Chile

**Keywords:** GPER-1, GPR30, G1 agonist, calcium signaling, tamoxifen resistance, kinin B1 receptor, breast cancer, cell proliferation

## Abstract

GPER-1 is a novel membrane sited G protein-coupled estrogen receptor. Clinical studies have shown that patients suffering an estrogen receptor α (ERα)/GPER-1 positive, breast cancer have a lower survival rate than those who have developed ERα-positive/GPER-1 negative tumors. Moreover, absence of GPER-1 improves the prognosis of patients treated with tamoxifen, the most used selective estrogen receptor modulator to treat ERα-positive breast cancer. MCF-7 breast cancer cells were continuously treated with 1,000 nM tamoxifen for 7 days to investigate its effect on GPER-1 protein expression, cell proliferation and intracellular [Ca^2+^]*i* mobilization, a key signaling pathway. Breast cancer cells continuously treated with tamoxifen, exhibited a robust [Ca^2+^]*i* mobilization after stimulation with 1,000 nM tamoxifen, a response that was blunted by preincubation of cells with G15, a commercial GPER-1 antagonist. Continuously treated cells also displayed a high [Ca^2+^]*i* mobilization in response to a commercial GPER-1 agonist (G1) and to estrogen, in a magnitude that doubled the response observed in untreated cells and was almost completely abolished by G15. Proliferation of cells continuously treated with tamoxifen and stimulated with 2,000 nM tamoxifen, was also higher than that observed in untreated cells in a degree that was approximately 90% attributable to GPER-1. Finally, prolonged tamoxifen treatment did not increase ERα expression, but did overexpress the kinin B1 receptor, another GPCR, which we have previously shown is highly expressed in breast tumors and increases proliferation of breast cancer cells. Although we cannot fully extrapolate the results obtained *in vitro* to the patients, our results shed some light on the occurrence of drug resistance in breast cancer patients who are ERα/GPER-1 positive, have been treated with tamoxifen and display low survival rate. Overexpression of kinin B1 receptor may explain the increased proliferative response observed in breast tumors under continuous treatment with tamoxifen.

## Introduction

Breast cancer is the most common type of cancer that produces high mortality in women, worldwide. In general, breast cancer is classified as estrogen receptor alpha (ERα) positive or negative. ERα-positive tumors comprise approximately 70% of all breast tumors and depend on estrogen to develop and grow (1, 2). It has been estimated that a large number of the responses mediated by 17β-estradiol, a kind of estrogen, occur through its binding to ERα, triggering a “genomic response” that initiates the transcription of genes associated to cell proliferation, survival and migration (1, 3). Nevertheless, estrogen also promotes a “rapid cellular response” (4), which includes an increase in intracellular calcium and activation of ERK1/2 mitogen-activated protein kinases (MAPKs), a signaling pathway that is considered crucial for cell proliferation (5, 6). Therefore, the efforts made so far to reduce breast cancer progress aim to suppress the synthesis of endogenous estrogen or to block ERα, through the use of selective estrogen modulators (SERMs), among which tamoxifen stands out (3). However, the molecular heterogeneity of breast cancer, together with the existence of more aggressive forms of the disease and resistance to conventional drug therapy, suggest that other players may be involved in the pathogenesis and progress of this neoplasia.

G protein-coupled estrogen receptor-1 (GPER-1 or GPR30) is a G protein-coupled receptor (GPCR) sited in the cell membrane that triggers a broad range of biological activities in response to stimulation by endogenous estrogens or dietary phytoestrogens (2, 7). Its gene is located on chromosome 7p22.3 and encodes a protein of 375 amino acids with a theoretical molecular mass of 41 kDa that is ubiquitously expressed in a large number of tissues (8–11). GPER-1 is highly expressed in the nervous and adipose tissues, liver and in the circulatory and immune systems among others. Its activation by 17β-estradiol has been corroborated by the use of labeled estradiol, and its synthetic agonist (G1) complemented with its pharmacological antagonist (G15) in normal and cancerous tissues and in various cell lines that do not express ERα (12, 13). GPER-1 mRNA has been detected in several breast cancer cell lines and its expression has been associated with the increased proliferation rate exhibited by these cells. GPER-1 signaling involves cAMP production and Ca^2+^ mobilization most likely through protein Gαs (13) and Src activation through Gβ*γ* (14) and the subsequent shedding of heparin-binding EGF-like growth factor (HB-EGF) and transactivation of epidermal growth factor receptor (EGFR). GPER-1 induces also the activation of phospholipase C and cFos and various kinases such as ERK1/2 MAPK, phosphoinositide 3-kinase/protein kinase B (PI3K/Akt) (6, 15–17). Evidence suggests that many of the responses attributed to ERα can be mediated, at least in part, by GPER-1. In fact, several of the beneficial responses produced by estrogens are absent in GPER-1 knockout mice (18, 19).

It has been shown that approximately 60% of all breast tumors are GPER-1-positive. In addition, expression of GPER-1 correlated with over-expression of HER-2, EGFR (HER-1), and lymph node status. Surprisingly, GPER-1 was negatively correlated with relapse-free survival in patients that were treated with tamoxifen compared to those receiving aromatase inhibitors (20–23). Surprisingly, independent studies have shown that tamoxifen and 4-OH tamoxifen (the main tamoxifen metabolite), two ERα antagonists, act as GPER-1 agonists (17, 22, 24). Furthermore, GPER-1 expression seems to be a favorable factor for relapse-free survival, but only in patients that did not receive tamoxifen; consequently, loss of GPER-1 improves the prognosis in patients treated with tamoxifen indicating that GPER-1 might be related to tamoxifen resistance in breast cancer (25). Activation of GPER-1 by 4-OH tamoxifen also increases the expression of connective tissue growth factor (CTGF), which may be related to a more aggressive behavior of some breast tumors (26).

In general, it is estimated that resistance mechanisms are related to mutations that arise within the intermediates that are part of the signaling pathways triggered by estradiol or its metabolites, promoting the survival and proliferation of tumor cells (27). Isolated models like those using tamoxifen-resistant MCF-7 cells (a cellular model that imitates therapeutic conditions), stimulated with estradiol point to an overexpression of GPER-1 (20). These observations showed that tamoxifen could act as non-specific GPER-1 agonist increasing breast cancer cells proliferation and migration. Moreover, it has recently been reported that patients with GPER-1-positive breast tumors, after four to six months of treatment with tamoxifen, not only generated resistance to therapy, but also suffered an increase in the size of tumor mass (28).

The current experiments were designed to examine the protein levels of GPER-1 in ERα-positive breast cancer cells that were continuously treated with tamoxifen for a period of 7 days and to investigate the mobilization of intracellular Ca^2+^ and cell proliferation that follows their stimulation with tamoxifen or GPER-1 agonists. We also investigated the protein levels of classical ERα and kinin B1 receptor (B1R), another GPCR associated to breast cancer progression (6, 29).

## Materials and Methods

### Cell Culture

MCF-7 cells, an estrogen-sensitive or ERα-positive/GPER-1-positive breast cancer cell line was used for all experiments. The MCF-7 cell line was obtained from the American Type Culture Collection (Manassas, VA USA). Cells were grown in modified Eagle’s Dulbecco (DMEM) supplemented with 10% fetal bovine serum (FBS), 2 mM glutamine and penicillin-streptomycin (10,000 U/ml sodium penicillin G and 10,000 μg/ml streptomycin sulfate; GIBCO BRL, Life Technologies) and 250 μg/ml fungizone. Cells were cultured at 37°C in a humidified incubator under 5% CO_2_ and 95% air (6, 29).

### Prolonged Exposure of Breast Cancer Cells to Tamoxifen

MCF-7 cells were grown and expanded for 7 days as mentioned above, in the presence of 1,000 nM tamoxifen (Sigma-Aldrich, USA). Medium containing tamoxifen was replaced every 48 h. After 7 days of exposure to tamoxifen, cells were frozen in cell culture freezing medium containing dimethyl sulfoxide (GIBCO BRL, Life Technologies) and stored until used. When required, these cells were grown again in a medium containing 1,000 nM tamoxifen (24). In parallel experiments, control cells that were not treated with the drug were grown as described above. Once a confluence of 80% was reached, both tamoxifen-treated and untreated cells were maintained for 24 h in culture medium without phenol red, and FBS. Once synchronized, tamoxifen-treated and untreated control cells were stimulated with 1,000 nM tamoxifen for 24, 48, and/or 72 h.

### Western Blotting

Cells were homogenized with cold RIPA buffer (5 mM Tris-HCl pH 7.4 containing 1 mM EDTA, 10 μg/ml aprotinin, 1 mM phenylmethane-sulphonyl fluoride, 1 μg/ml leupeptin, and 10 μg/ml pepstatin). Proteins were separated by sodium dodecyl sulphate-polyacrylamide gel electrophoresis (SDS-PAGE) and transferred onto Immobilon-P membranes (Millipore, Billerica, MA USA). Membranes were incubated with primary antibodies for 2 h and then with the corresponding peroxidase-labeled secondary antibody for 30 min. Peroxidase activity was visualized using a commercial chemiluminescence kit (Pierce, Rockford, USA). Anti-GPER-1 is a rabbit polyclonal antibody directed to the C-terminus of the human receptor (ab39742; Abcam, UK). Furthermore, antibodies raised against ERα (PA5-16440; Invitrogen, USA) and the kinin B1R (6) were used. The antibodies used for the first immunodetection procedure were stripped off as previously described (6) and glyceraldehyde 3-phosphate dehydrogenase (GAPDH, Millipore) was then detected as control of protein loading.

### Measurement of the [Ca^2+^]*_i_*

Cells were grown and synchronized in medium without phenol red, FBS and antibiotics for 48 h. Then, they were gently trypsinized, washed with PBS and incubated for 30 min at 37°C at a concentration of 5 × 10^6^ cells/ml, in the darkness with 5 mM Indo-1 AM, a ratiometric fluorescent probe (Life Technologies). Next, cells were washed with PBS, resuspended in 25 mM Hepes buffer pH 7.4 containing (125 mM NaCl, 5 mM KCl, 1 mM CaCl_2_, 0.5 mM MgCl_2_, 1 mM NaH_2_PO4, 0.1% bovine serum albumin and 0.1% glucose) (6, 30). During the assay, cells were stimulated with 100 nM ATP, which was used as a positive control. Additional controls were performed by using the anionic detergent triton X-100 and the calcium-chelating agent, EGTA (**Figure 3C**). Following stimulation with ATP, cells were stimulated with a range of concentrations of 17β-estradiol (0.1, 1 and 10 nM) (Sigma-Aldrich, Germany) or G1, the synthetic GPER-1 agonist (1, 10 and 100 nM) (Tocris, USA). Additionally, cells were pretreated for 5 min with an excess (1,000 nM) of the GPER-1 antagonist, G15 (Tocris, USA) prior stimulation with 17β-estradiol or G1. Both, cells under prolonged treatment with tamoxifen (tamoxifen-treated cells) and untreated cells were stimulated with 10 nM 17β-estradiol, 100 nM G1 or 1,000 nM tamoxifen. Tamoxifen was dissolved in ethanol and the other drugs were solubilized in dimethyl sulfoxide (Sigma-Aldrich, Germany).

Measurements were carried out in a spectrofluorometer LS55 (Perkin Elmer, Wellesley, MA USA) using 2 × 10^5^ cells/ml in a thermostated cuvette that was under continuous agitation. Measurements were performed using an excitation wavelength of 330 nm, and emissions of 405 nm and 480 nm were recorded. Data were expressed using the ratiometric relationship between the absorbance at 405 and 480 nm (30).

### Cell Proliferation Assay

Cells were seeded on 96-well plates and cultured at 70% subconfluence. After synchronization for 48 h in phenol red-free DMEM without FBS (6), cells were stimulated with a range of concentrations of tamoxifen (50, 100, 500, 1,000, and 2,000 nM). Comparative experiments were performed on cells stimulated, under identical conditions, but following pretreatment with the GPER-1 antagonist G15. After 24 h under stimulation, cells were pulsed with 5-bromo-2’-deoxyuridine (BrdU) for 2 h. Incorporation of BrdU was determined by a colorimetric immunoassay according to manufacturer’s protocol (Roche, Germany). At least three independent experiments were carried out for each concentration point and each point was performed in triplicates. Positive control experiments were carried out stimulating cells with 10% FBS.

### Statistical Analysis

Statistical evaluation between experimental groups was done with ANOVA analysis followed by post-test pairwise comparisons using Tukey’s test. Values were expressed as mean ± SEM and significance was considered acceptable at the 5% level (P < 0.05).

## Results

### Prolonged Tamoxifen Treatment Induces GPER-1 Overexpression and Increases Breast Cancer Cells Proliferation Rate

**Figure 1:**
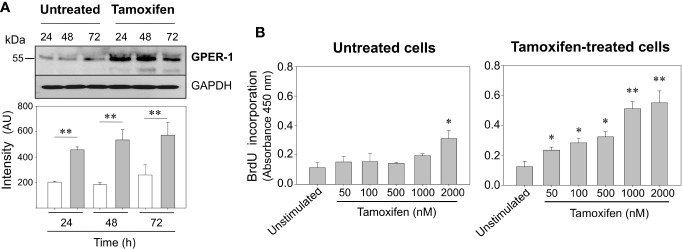
Continuous tamoxifen treatment induces GPER-1 overexpression and an increase in BrdU incorporation in response to stimulation with tamoxifen. **(A)** MCF-7 cells that were under treatment with tamoxifen for 7 days together with their respective untreated controls were cultured, synchronized and then stimulated with 1,000 nM tamoxifen for 24, 48 and 72 h. Cells were homogenized and proteins separated by SDS-PAGE, transferred onto Immobilon-P membranes and immunoblotted using a specific antibody for GPER-1. The antibody was stripped off and the same membrane incubated with anti-GAPDH antibody as loading control. Data are representative of two independent experiments (n = 2). **P < 0.001. **(B)** Untreated cells or continuously exposed to tamoxifen were grown on 96-well plates, synchronized and stimulated with various concentrations of tamoxifen for 24 h. BrdU incorporation was determined by a colorimetric cell proliferation immunoassay and measuring absorbance at 450 nm. Results are shown as mean ± SEM (n = 3) *P < 0.05; **P < 0.001 between stimulated and unstimulated cells in both tamoxifen-treated and untreated cells.

It has been shown that use of tamoxifen on patients with initial GPER-1 positive breast tumors increases GPER-1 protein expression and markedly reduces patient survival (20). Here we have corroborated that GPER-1 is overexpressed in breast cancer cells exposed for 7 days to 1,000 nM tamoxifen (**Figure 1A**), a concentration similar to that found in breast tissue of patients treated with this drug (24). When untreated cells and those under continuous treatment with tamoxifen were challenged with 1,000 nM tamoxifen for 24, 48, or 72 h, a significant increase in the expression of GPER-1 was observed in the cells under continuous treatment with the drug (**Figure 1A**). Furthermore, the proliferation rate, assessed as BrdU incorporation, in the cells that were under prolonged treatment with tamoxifen was significantly higher than those that were not under treatment (**Figure 1B**). A concentration-dependent response was clearly observed when the cells under prolonged treatment were stimulated with tamoxifen ranging 50 to 2,000 nM (**Figure 1B**). By comparison, cells that were not under continuous treatment with tamoxifen showed an increase in BrdU incorporation only when they were stimulated with 2,000 nM tamoxifen (**Figure 1B**).

### Prolonged Tamoxifen Treatment Increases Intracellular Calcium Signaling in Breast Cancer Cells Challenged With Tamoxifen

**Figure 2:**
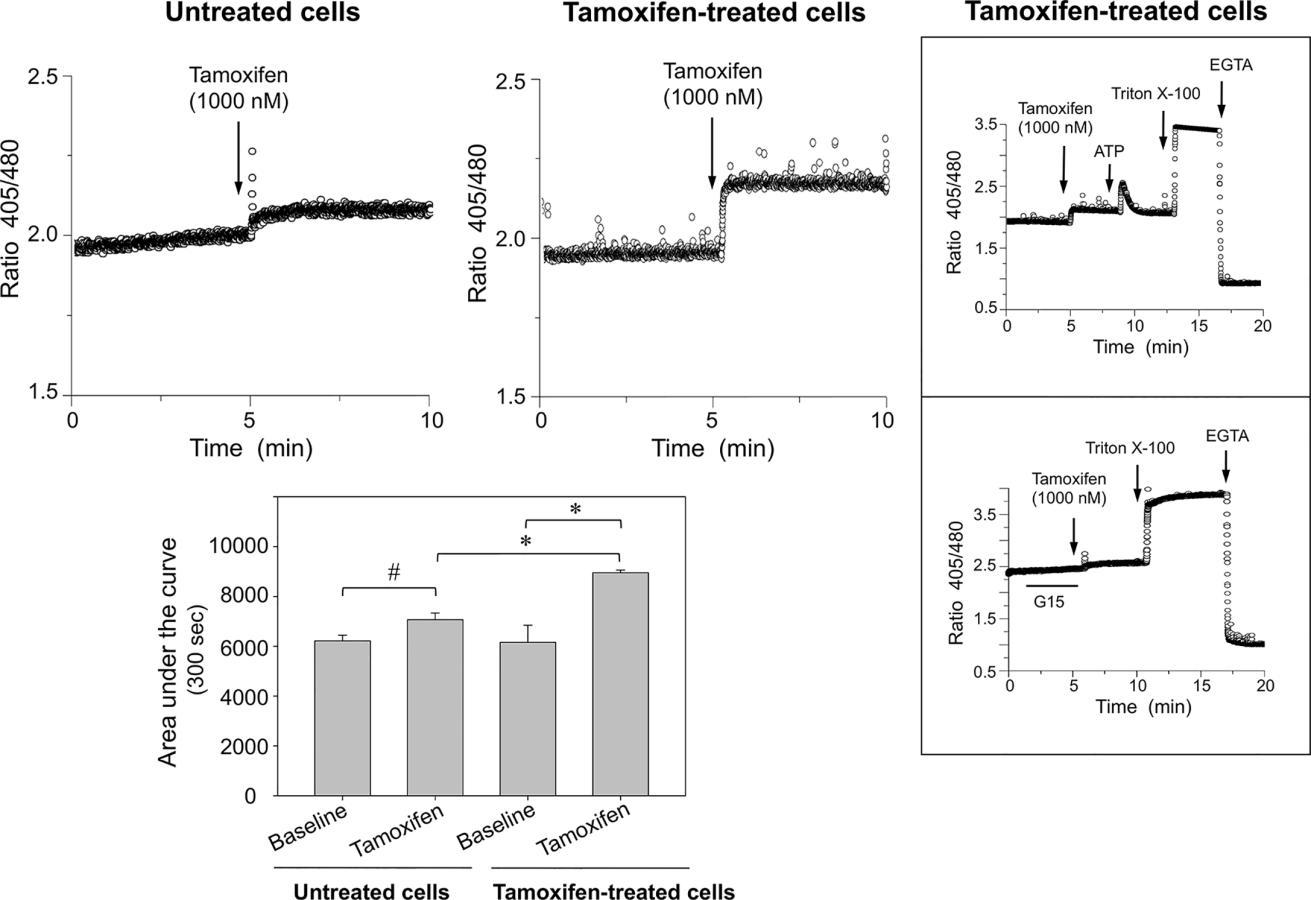
Breast cancer cells continuously exposed to tamoxifen display higher [Ca^2+^]*_i_* mobilization than untreated controls following stimulation with tamoxifen. MCF-7 cells that were under prolonged treatment with tamoxifen for 7 days, together with their respective untreated controls, were cultured, detached, and loaded with Indo-AM calcium probe and stimulated with 1,000 nM tamoxifen. The area under the curve was estimated to assess the magnitude of the response in both conditions. Results are shown as mean ± SEM (n = 3); *P < 0.05; ^#^Not significant. *Insert*: The response elicited by tamoxifen was inhibited by preincubation of cells for 5 min with 1,000 nM G15, a GPER-1 antagonist. Additional controls using ATP, triton X-100 and EGTA are also shown.

To examine whether continuous tamoxifen exposure modifies intracellular calcium signaling, cells were synchronized before labeling with the Indo-1 AM probe. Following a pulse with 1,000 nM tamoxifen, a significant [Ca^2+^]*_i_* mobilization was generated in cells under prolonged tamoxifen treatment when compared with those that were not under prolonged treatment; in the latter, [Ca^2+^]*_i_* mobilization was almost negligible (**Figure 2**). Cell integrity was assessed by stimulation of the same cells with a pulse of 100 nM ATP (**Figure 2**, top inbox). This response was comparable to that obtained in cells that were not under prolonged treatment with tamoxifen and were also stimulated with 100 nM ATP (not shown). Further controls of the technique included addition of triton X-100 and EGTA. Interestingly, preincubation of cells continuously treated with tamoxifen, for 5 min with 1,000 nM G15 (GPER-1 antagonist) significantly reduced the [Ca^2+^]*_i_* mobilization triggered by tamoxifen in these cells, suggesting that an important fraction of the response to tamoxifen may be due to GPER-1 activation (**Figure 2**, bottom inbox).

### Breast Cancer Cells Continuously Exposed to Tamoxifen Display Higher [Ca^2+^]*_i_* Mobilization Than Untreated Cells When Stimulated With Estrogen or G1

**Figure 3:**
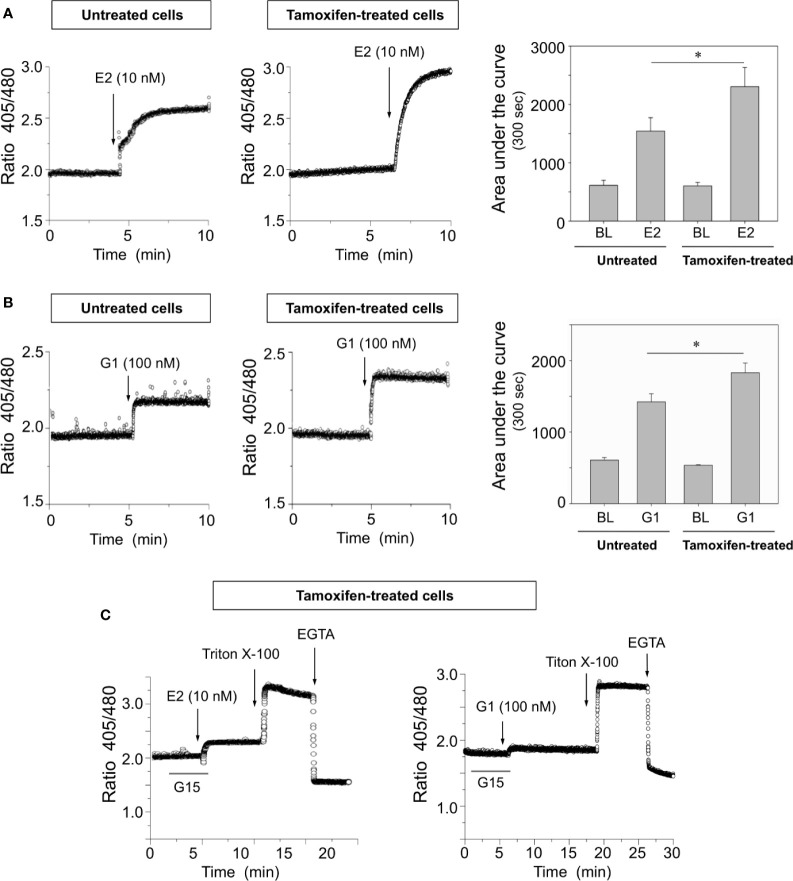
Breast cancer cells continuously exposed to tamoxifen exhibit higher [Ca^2+^]*_i_* mobilization than untreated controls when are stimulated with 17β-estradiol or G1. Continuously treated and untreated MCF-7 cells were cultured, detached and loaded with Indo-AM calcium probe before stimulation with 10 nM 17β-estradiol (E2) **(A)** or 100 nM G1 **(B)**, a specific synthetic agonist of GPER-1. The area under the curve was calculated and graphed to assess the magnitude of the response in each condition. BL, baseline. **(C)** Preincubation for 5 min with 1,000 nM G15 significantly decreased [Ca^2+^]*_i_* mobilization in tamoxifen-treated cells; additional controls using triton X-100 and EGTA are also depicted. Results are shown as mean ± SEM (n = 3) *P < 0.05; between tamoxifen-treated cells and untreated controls.

To examine the influence of ERα and GPER-1 in calcium signaling of breast cancer cells, several approaches were carried out. These experiments showed that cells continuously treated with tamoxifen exhibited higher [Ca^2+^]*_i_* mobilization than untreated cells when they were stimulated with 10 nM 17β-estradiol or 100 nM G1, a specific GPER-1 agonist (**Figures 3A, B**). The increase in [Ca^2+^]*_i_* mobilization in response to 17β-estradiol was approximately 50% greater than that produced by the cells which had not been under prolonged treatment with tamoxifen; by comparison the increase produced by G1 was approximately 25% higher than that observed in untreated cells (**Figures 3A, B**). Preincubation of continuously treated cells with 1,000 nM G15, a GPER-1 antagonist, blunted the response triggered by G1 (**Figure 3C**). Interestingly, the same antagonist significantly reduced the response generated by 17β-estradiol. Additional controls were performed by using the anionic detergent triton X-100 and the calcium-chelating agent EGTA (**Figure 3C**). These results suggest that the increase in [Ca^2+^]*_i_* mobilization, triggered by G1 or 17β-estradiol in tamoxifen-treated cells, is mediated mainly by GPER-1.

### Prolonged Tamoxifen Treatment Increases the Proliferation Rate of Breast Cancer Cells in Response to Estrogen, G1, and Tamoxifen

**Figure 4:**
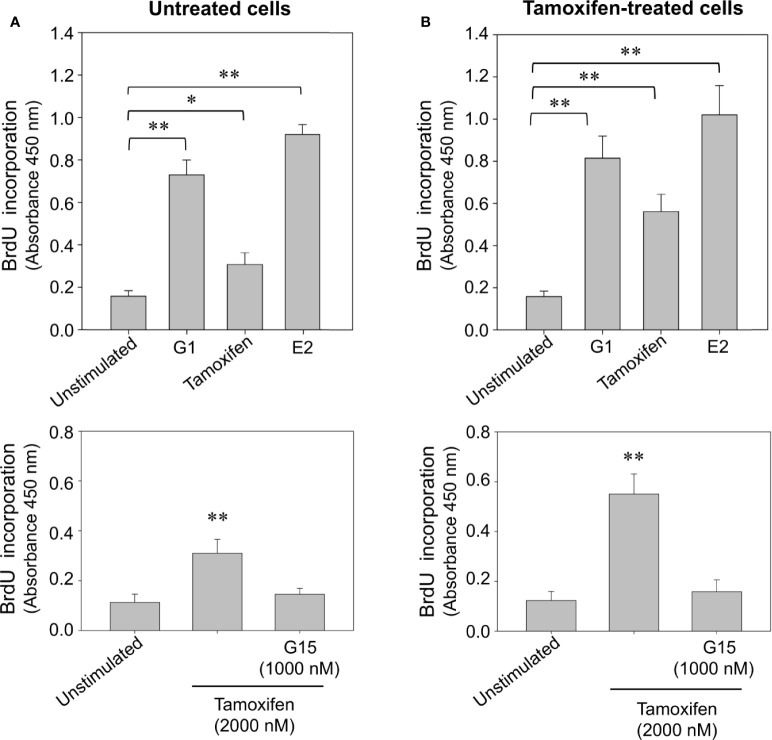
Continuous tamoxifen treatment induces an increase in BrdU incorporation in breast cancer cells stimulated with tamoxifen. Untreated cells **(A)** or continuously exposed to tamoxifen **(B)** were grown on 96-well plates, synchronized and stimulated with 2,000 nM tamoxifen, 10 nM 17β-estradiol (E2) or 100 nM G1 for 24 h. BrdU incorporation was determined by a cell proliferation immunoassay and by measuring absorbance at 450 nm. The effect produced by tamoxifen on BrdU incorporation was significantly reduced by preincubation of cells with 1,000 nM G15 in both, untreated cells and cells continuously exposed to the drug. Results are shown as mean ± SEM (n = 3) *P < 0.05; **P < 0.001 versus unstimulated cells or preincubated with G15.

To examine whether prolonged exposition of breast cancer cells to tamoxifen increases the cycling activity of these cells, a proliferation assay based on the incorporation of BrdU, an analogue of thymidine was performed. An increase in the incorporation of BrdU was observed in untreated and continuously treated cells stimulated for 24 h with 10 nM 17β-estradiol and 100 nM G1 (**Figure 4**). This increase was also significant when cells were stimulated with 2,000 nM tamoxifen (**Figure 4**). Interestingly, the increase in BrdU incorporation observed following stimulation with tamoxifen was inhibited by pretreatment of both continuously treated and untreated cells with G15, the GPER-1 antagonist (**Figures 4A, B**).

### Prolonged Tamoxifen Treatment Overexpresses the Kinin B1 Receptor but Not ERα in Breast Cancer Cells

**Figure 5:**
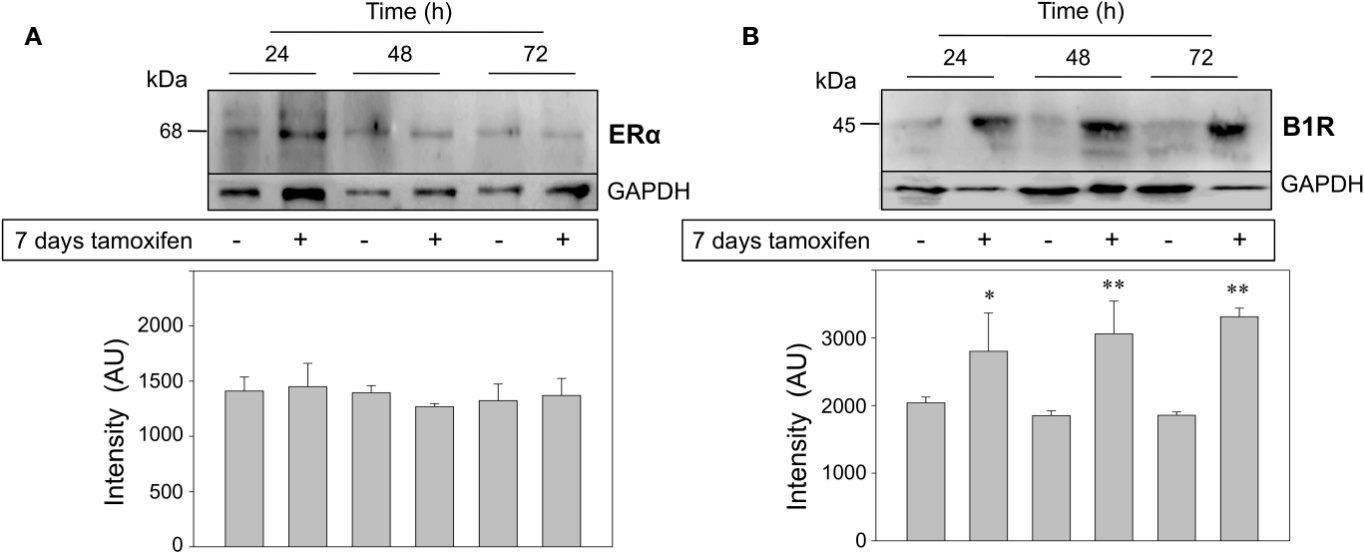
Breast cancer cells continuously treated with tamoxifen overexpress the kinin B1R, but not ERα. MCF-7 cells that were under treatment with tamoxifen for 7 days together with their respective untreated controls were stimulated with 1,000 nM tamoxifen by 24, 48, and 72 h. Cell proteins were separated by SDS-PAGE, transferred onto Immobilon-P and immunoblotted with antibodies for detection of ERα **(A)** and kinin B1R **(B)**. Antibodies were stripped off and the same membranes were incubated with an antibody directed to GAPDH as control for protein loading. Data are representative of two independent experiments (n = 2). *P < 0.05; **P < 0.001 between tamoxifen-treated and untreated cells.

Finally, we addressed two crucial questions: the first one was whether the treatment of MCF-7 cells with 1,000 nM tamoxifen for 7 days modified the expression levels of the classical estrogen receptor, ERα. As expected, protein expression levels of ERα did not change in tamoxifen-treated breast cancer cells respect to the untreated cells (**Figure 5A**). The second question was to examine the expression levels of another GPCR already known to favor the malignant phenotype of ERα positive breast cancer cells. Our previous studies have shown that stimulation of the kinin B1R results in an increase in cell proliferation, chemotaxis and release of matrix metalloproteases 2 and 9 from breast cancer cells (6, 29). Unexpectedly, kinin B1R protein expression was dramatically increased in the continuously treated cells that were additionally stimulated with 2,000 nM tamoxifen for 24, 48, and 72 h (**Figure 5B**), an effect that reinforces our first observations that kinin B1R stimulation increases the proliferation of breast cancer cells even at higher levels than estrogen (6). Our results suggest that ERα would not be directly involved in pharmacological resistance.

## Discussion

Estrogens, predominantly 17β-estradiol and its classical receptor, ERα, contribute to the development and progression of breast cancer. Drugs that block estrogen production or signaling by binding to ERα have been successfully used for many years. Such therapy includes SERMs (e.g., tamoxifen, raloxifene), antagonists of ERα (e.g., fulvestrant) and aromatase inhibitors, including reversible non-steroidal agents (e.g., letrozole, anastrozole), among others. Tamoxifen is, so far, one of the most commonly antiestrogenic drugs used for breast cancer treatment (31, 32). Endocrine therapy, based on the use of tamoxifen, has predominantly antiestrogenic effects in the breast and is aimed to block ERα in estrogen-sensitive breast cancer. Nevertheless, breast cancer patients may acquire resistance to antiestrogenic drugs complicating treatment. On the other hand, the existence of more complex and undiscovered signaling pathways beyond estrogen receptors appears to control cancer progression (33, 34). Thereby, the use of tamoxifen on breast cancer patients with initial GPER-1 positive tumors increased GPER-1 protein expression and markedly reduced survival (20).

The MCF-7 breast cancer cell line has emerged as one of the most widely used tool to scrutinize the effects of estrogen, SERMs and ERα antagonists. Although we cannot fully extrapolate the results obtained *in vitro* to the patients, this cell line is a good example of those mammary tumors made up of cells that express both ERα and GPER-1. Similarly, MCF-7 cells have been used to investigate the resistance to antiestrogenic drugs such as tamoxifen. Clearly, these studies should be expanded to other ERα positive breast cancer cell lines such as T47D and ZR-75-1 cells or better yet to 3D cultures using different cell lines or tumor cells directly obtained from patients with ERα positive breast tumors. Our experiments showed that MCF-7 cells exposed for 24 h to various concentrations of tamoxifen increased BrdU incorporation (DNA synthesis) when tamoxifen was present at 2,000 nM. A similar observation had been reported in the late eighties by Wakeling et al. (35), using also MCF-7 cells. Furthermore, Reddel and Sutherland (36) found that 10 nM tamoxifen had a proliferative effect on T47D breast cancer cells, a cell line which like MCF-7 cells expresses both ERα and GPER-1. Moreover, almost 50 years ago, tamoxifen had already been blamed to increase the growth of some types of breast cancer (37, 38). Interestingly, tamoxifen and 4-OH tamoxifen (the main metabolite of tamoxifen), two compounds that antagonize estrogen binding to ERα, are GPER-1 agonists (8, 17, 39). In a series of seminal experiments, Thomas et al. (13) were the first to describe a competitive binding (*K*_i_ values in the 10^-7^ M range) between estrogen and tamoxifen for GPER-1 expressed by SKBr3 breast cancer cells (ERα and ERβ negative, GPER-1 positive) or expressed by HEK cells transfected with GPER-1. Moreover, tamoxifen binding to GPER-1 resulted in activation of a stimulatory G protein and increase in adenylyl cyclase activity and cAMP levels. Subsequent experiments have shown that agonistic activity of tamoxifen or 4OH-tamoxifen triggers signaling pathways such as PI3K, ERK1/2 MAPK, and EGFR transactivation (14). The EGFR/ERK1/2 signaling cascade upregulates the expression of Egr-1 that in turn participates in the transcription of CTGF and cyclin D1, two genes that regulate breast cancer growth (16, 40). Similarly, agonistic activity of tamoxifen increases the proliferation of endometrial cancer cells by activating the GPER-1/EGFR/ERK1/2/CyclinD1 route, data that is in agreement with the observation that endometrial cancer patients under tamoxifen treatment exhibit a worse prognosis (41).

Previous reports have shown an increased translocation of GPER-1 to the cell surface of MCF-7 breast cancer cells that were continuously exposed to 10 nM tamoxifen for 6 months and stimulated with 17β-estradiol (20). Furthermore, other studies indicate that concentrations of tamoxifen and 4-OH tamoxifen reached in breast tissue of patients with ERα-positive breast cancer are significantly higher (up to about 100 times) than those present in plasma (42). Our results show that treatment of MCF-7 cells with 1,000 nM tamoxifen for 7 days produces a significant increase of GPER-1 protein expression. It is important to point out that this concentration is similar to that found in breast tissue of breast cancer patients treated with the drug (24). Other studies have shown that after 12 months of treatment with 10^−7^ M tamoxifen, this drug no longer inhibits the proliferative effect of estrogen on MCF-7 cells; during this period of time, tamoxifen did not increase cell proliferation (43). Furthermore, long-term exposure to tamoxifen has been shown to increase aromatase expression and activity, effects that depend on GPER-1 activity (44). Our results indicate that a short period of 7 days under continuous treatment with 1,000 nM tamoxifen induces overexpression of GPER-1, making breast cancer cells more sensitive to tamoxifen, which following GPER-1 activation triggers DNA synthesis, an effect that can be blocked by a specific GPER-1 antagonist. Therefore, overexpression and activity of GPER-1 appear as crucial steps for tamoxifen resistance since tamoxifen could increase cell proliferation directly by stimulating GPER-1 or indirectly by rising estrogen levels as result of an increase in the activity and expression of aromatase.

GPER-1 overexpression could be associated to carcinogenesis and to molecular strategies developed by tumor cells to escape tamoxifen treatment. GPER-1 overexpression has been observed in invasive ductal carcinomas of the breast when compared to adjacent healthy tissue (23) and in inflammatory breast cancer, a more aggressive form of this neoplasia (45). Signaling through GPER-1 has been shown to trigger the expression of IL-1β and IL-1R1 in cancer-associated fibroblasts and breast cancer cells, respectively. Thus, coupling of IL1β secretion by cancer-associated fibroblasts to the expression of IL-1R1 by cancer cells, promotes a positive regulation of protumoral genes such as those for COX-2 and ATP-binding cassette super-family G member 2 (46). Yu et al., 2020 (47) reported that ERα positive metastatic tissue shows increased levels of GPER-1 and ATP-binding cassette super-family G member 2 genes, which have been involved with multiresistance to different types of chemotherapy. Additionally, treatment of tamoxifen-resistant MCF-7 cells with G1 or with Fulvestrant (ICI 182,780) significantly increased GPER-1 expression, when compared to non-resistant MCF-7 cells (47). Pharmacological therapies can also alter intracellular signaling cascades such as [Ca^2+^]*_i_* signaling, a key pathway in which calcium itself acts as second messenger or may participate in signal transduction to open ion channels. However, few studies have addressed the release of intracellular calcium triggered by tamoxifen in breast cancer cells. Our experiments showed that after prolonged treatment with tamoxifen, breast cancer cells stimulated with 1,000 nM tamoxifen mobilized [Ca^2+^]*_i_* whereas untreated cells did not generate such a response. This response is a result of GPER-1 overexpression attributed to the prolonged treatment of breast cancer cells with the drug. In fact, preincubation of tamoxifen-treated cells with 1,000 nM G15 reduced [Ca^2+^]*_i_* to basal levels. Interestingly, [Ca^2+^]*_i_* was also increased when continuously treated cells were stimulated with 10 nM 17β-estradiol, an effect that was greatly reduced following preincubation of cells with the GPER-1 antagonist suggesting that GPER-1 may also be involved in this response. GPER-1 overexpression was further manifested when continuously treated cells were stimulated with 100 nM G1, a synthetic GPER-1 agonist that also increased [Ca^2+^]*_i_* in these cells.

Relevance of [Ca^2+^]*_i_* mobilization in breast cancer cells has recently been adressed by Ji et al. (48) who showed that Cav1.3 (a subunit of the L-type calcium channel) is widely expressed in breast cancer tissue and is upregulated by estrogen. Notably, silencing of GPER-1 inhibited the positive regulation of Cav1.3 induced by estrogen, reversing the increase in intracellular calcium release and proliferation of breast cancer cells. Our experiments indicate that breast cancer cells continuously treated with tamoxifen exhibited a concentration-dependent increase in BrdU incorporation after stimulation with various concentrations of tamoxifen. As expected, preincubation of cells with 1,000 nM G15 reduced the BrdU incorporation induced by tamoxifen. Although the mechanisms of resistance in estrogen-sensitive breast cancer are probably multifactorial, our evidence indicates that at least part of the phenomenon may be due to overexpression and activation of GPER-1. This observation may be of great relevance for breast cancer patients that suffer from breast tumors that co-express GPER-1 and ERα and undergo tamoxifen treatment.

GPCRs are preponderant for tumor development, progression and generation of drug resistance (32). Therefore, we explored the possibility of molecular interactions between GPER-1 and the kinin B1R, another member of the GPCR family, which is strongly expressed in ERα positive breast cancer cells. We have previously shown that kinin B1R favors the malignant phenotype of breast cancer cells (MCF-7, T47D, and ZR-75-1 cells) because its stimulation by B1R agonists induces cell proliferation and secretion of metalloproteinases-2 and -9, and of kallikrein-related peptidase 6, among other effects (6, 29, 49). Coincidently, kinin B1R agonists are at higher levels in serum of patients with breast cancer than in healthy subjects (50), an observation that matched the presence of kinin B1R binding sites detected by our group in fibroadenomas, ductal carcinomas *in situ* and in invasive ductal carcinomas (6). Furthermore, the use of inhibitors has shown that metalloproteinases secretion and proliferation of breast cancer cells relies on EGFR transactivation and activation of the EGFR/ERK1/2 MAPK cascade (6, 28).

Interestingly, we observed B1R overexpression in breast cancer cells that were under exposure to tamoxifen for a 7-day period. Our data suggest that kinin B1R overexpression is an early event and that together with GPER-1 it may be part of a cross-talk network in estrogen-sensitive breast cancer cells to enhance cell proliferation and/or metastasis by activating signaling mechanisms, which are independent of ERα. Analysis of data from a subset of breast cancer patients has shown that GPER-1 expression has also been positively correlated with overexpression of EGFR (21). Since, a cooperative effect or association between GPER-1 and kinin B1R in breast cancer has not been explored yet; a next set of experiments should be focused on the role of both receptors in breast cancer patients. In addition, the GPER-1/EGFR signaling axis mediates the expression of cell cycle regulatory genes in cancer-associated fibroblasts derived from breast cancer patients, favoring tumor progression (40). It has recently been shown that stimulation with tamoxifen, activates GPER-1, improving breast cancer stem cells viability and stemness and BAD phosphorylation, event that seems to be an alternative survival mechanism for these cells (51).

Together our findings suggest that GPER-1 plays a key role in the mechanisms of tamoxifen resistance of estrogen-sensitive breast cancer cells, extending the limits of understanding of the effects generated by tamoxifen in these cells.

## Data Availability Statement

The raw data supporting the conclusions of this article will be made available by the authors, without undue reservation.

## Author Contributions

LM contributed to the design and execution of the experiments, writing, discussion, and revision of the article. PE and CF contributed to the experimental design, discussion, writing, and revision of the article. FB, AO, and IR contributed to the experimental procedures.

## Funding

The authors wish to thank FONDECYT for grant 1201635 (PE) and Vicerrectoría de Investigación, Desarrollo y Creación Artistica from Universidad Austral de Chile and the Departamento de Ciencias Básicas, Facultad de Medicina y Ciencia, Universidad San Sebastián for its continuous support.

## Conflict of Interest

The authors declare that the research was conducted in the absence of any commercial or financial relationships that could be construed as a potential conflict of interest.
